# Identification of post-cardiac arrest blood pressure thresholds associated with outcomes in children: an ICU-Resuscitation study

**DOI:** 10.1186/s13054-023-04662-9

**Published:** 2023-10-07

**Authors:** Monique M. Gardner, David A. Hehir, Ron W. Reeder, Tageldin Ahmed, Michael J. Bell, Robert A. Berg, Robert Bishop, Matthew Bochkoris, Candice Burns, Joseph A. Carcillo, Todd C. Carpenter, J. Michael Dean, J. Wesley Diddle, Myke Federman, Richard Fernandez, Ericka L. Fink, Deborah Franzon, Aisha H. Frazier, Stuart H. Friess, Kathryn Graham, Mark Hall, Monica L. Harding, Christopher M. Horvat, Leanna L. Huard, Tensing Maa, Arushi Manga, Patrick S. McQuillen, Kathleen L. Meert, Ryan W. Morgan, Peter M. Mourani, Vinay M. Nadkarni, Maryam Y. Naim, Daniel Notterman, Murray M. Pollack, Anil Sapru, Carleen Schneiter, Matthew P. Sharron, Neeraj Srivastava, Bradley Tilford, Shirley Viteri, David Wessel, Heather A. Wolfe, Andrew R. Yates, Athena F. Zuppa, Robert M. Sutton, Alexis A. Topjian

**Affiliations:** 1 Department of Anesthesiology and Critical Care Medicine, The Children’s Hospital of Philadelphia, University of Pennsylvania, 3401 Civic Center Boulevard, Philadelphia, PA 19104 USA; 2 Department of Pediatrics, University of Utah, Salt Lake City, UT USA; 3 Department of Pediatrics, Children’s Hospital of Michigan, Central Michigan University, Detroit, MI USA; 4 Department of Pediatrics, Children’s National Hospital, George Washington University School of Medicine, Washington, DC USA; 5 Department of Pediatrics, University of Colorado School of Medicine and Children’s Hospital Colorado, Aurora, CO USA; 6 Department of Critical Care Medicine, UPMC Children’s Hospital of Pittsburgh, University of Pittsburgh, Pittsburgh, PA USA; 7 Department of Pediatrics and Human Development, Michigan State University, Grand Rapids, MI USA; 8 Department of Pediatrics, Mattel Children’s Hospital, University of California Los Angeles, Los Angeles, CA USA; 9 Department of Pediatrics, Nationwide Children’s Hospital, The Ohio State University, Columbus, OH USA; 10 Department of Pediatrics, Benioff Children’s Hospital, University of California, San Francisco, San Francisco, CA USA; 11 Nemours Cardiac Center, Nemours Children’s Health and Thomas Jefferson University, Wilmington, DE USA; 12 Department of Pediatrics, Washington University School of Medicine, St. Louis, MO USA; 13 Department of Pediatrics, University of Arkansas for Medical Sciences and Arkansas Children’s Hospital, Little Rock, AR USA; 14 Department of Molecular Biology, Princeton University, Princeton, NJ USA; 15 Department of Pediatrics, Nemours Children’s Health and Thomas Jefferson University, Wilmington, DE USA

**Keywords:** Pediatric, Infant, Neonatal, Cardiopulmonary resuscitation, Post-cardiac arrest, Blood pressure, Hypotension, Outcomes

## Abstract

**Introduction:**

Though early hypotension after pediatric in-hospital cardiac arrest (IHCA) is associated with inferior outcomes, ideal post-arrest blood pressure (BP) targets have not been established. We aimed to leverage prospectively collected BP data to explore the association of post-arrest BP thresholds with outcomes. We hypothesized that post-arrest systolic and diastolic BP thresholds would be higher than the currently recommended post-cardiopulmonary resuscitation BP targets and would be associated with higher rates of survival to hospital discharge.

**Methods:**

We performed a secondary analysis of prospectively collected BP data from the first 24 h following return of circulation from index IHCA events enrolled in the *ICU-RESUScitation* trial (NCT02837497). The lowest documented systolic BP (SBP) and diastolic BP (DBP) were percentile-adjusted for age, height and sex. Receiver operator characteristic curves and cubic spline analyses controlling for illness category and presence of pre-arrest hypotension were generated exploring the association of lowest post-arrest SBP and DBP with survival to hospital discharge and survival to hospital discharge with favorable neurologic outcome (Pediatric Cerebral Performance Category of 1–3 or no change from baseline). Optimal cutoffs for post-arrest BP thresholds were based on analysis of receiver operator characteristic curves and spline curves. Logistic regression models accounting for illness category and pre-arrest hypotension examined the associations of these thresholds with outcomes.

**Results:**

Among 693 index events with 0–6 h post-arrest BP data, identified thresholds were: SBP > 10th percentile and DBP > 50th percentile for age, sex and height. Fifty-one percent (*n* = 352) of subjects had lowest SBP above threshold and 50% (*n* = 346) had lowest DBP above threshold. SBP and DBP above thresholds were each associated with survival to hospital discharge (SBP: aRR 1.21 [95% CI 1.10, 1.33]; DBP: aRR 1.23 [1.12, 1.34]) and survival to hospital discharge with favorable neurologic outcome (SBP: aRR 1.22 [1.10, 1.35]; DBP: aRR 1.27 [1.15, 1.40]) (all *p* < 0.001).

**Conclusions:**

Following pediatric IHCA, subjects had higher rates of survival to hospital discharge and survival to hospital discharge with favorable neurologic outcome when BP targets above a threshold of SBP > 10th percentile for age and DBP > 50th percentile for age during the first 6 h post-arrest.

**Supplementary Information:**

The online version contains supplementary material available at 10.1186/s13054-023-04662-9.

## Introduction

More than 15,000 children per year in the USA have an in-hospital cardiac arrest (IHCA), with survival rates of 45–50% [1, 2]. Children who survive the cardiac arrest event are at high risk for post-cardiac arrest syndrome (PCAS), which includes brain injury, myocardial dysfunction, ischemia and reperfusion injury, and the ongoing underlying precipitating condition [3]. Hypotension, which is common during PCAS, is due to a combination of myocardial dysfunction and the ischemia–reperfusion syndrome [4] and can lead to secondary cerebral and myocardial injury resulting in worse outcomes.

Post-cardiac arrest systolic hypotension has previously been defined as systolic blood pressure (SBP) less than the 5th percentile for age and sex based on normative data of blood pressures in the healthy pediatric population [5]. Prior studies in both adults [6–10] and pediatrics [11–14] have focused on early post-arrest blood pressure measurements (i.e., within the first 6 h after return of spontaneous circulation [ROSC]) when organ reperfusion may be particularly critical and have demonstrated that early hypotension is associated with higher rates of post-arrest hospital mortality and worse neurologic outcomes. Consequently, pediatric guidelines recommend avoiding blood pressure less than the 5th percentile for age in the post-arrest period [3, 15], though this cutoff was selected without prior evidence, and no previous study has sought to identify best blood pressure thresholds in pediatrics. In contrast, adult studies have established that survival to hospital discharge is associated with minimum blood pressure thresholds much higher than the 5th percentile [16] and that myocardial injury can be mitigated with post-arrest mean arterial pressure (MAP) > 80 mmHg [17].

Using prospectively collected data from the *ICU-RESUScitation* (*ICU-RESUS*) clinical trial [18, 19], we aimed to define post-arrest SBP and diastolic blood pressure (DBP) percentile thresholds based on the probability of survival to hospital discharge with favorable neurologic outcome and then evaluate the association of these new thresholds at 0–6 h and 6–24 h post-arrest with survival to hospital discharge and survival to hospital discharge with favorable neurologic outcome. In a subset of subjects with intra-arrest hemodynamic data, we aimed to evaluate the association of intra-arrest BP targets with post-arrest BP thresholds.

## Methods

The *ICU-RESUS* trial (ClinicalTrials.gov Identifier: NCT02837497) was a parallel-stepped-wedge hybrid cluster-randomized interventional trial in 18 pediatric intensive care units and pediatric cardiac intensive care units (ICUs, clusters) from 10 clinical sites in the USA between October 1, 2016, and March 31, 2021. The methods [18] and primary results [19] have been previously published. Inclusion criteria were age between 37 weeks corrected gestation and ≤ 18 years and CPR of any duration in the ICU. Subjects were excluded if goals of care limited aggressive ICU therapies prior to arrest, they were brain dead, or they had an out-of-hospital cardiac arrest associated with the current hospitalization. The intervention included a two-part ICU resuscitation quality improvement bundle of point-of-care cardiopulmonary resuscitation (CPR) training on a manikin and structured cardiac arrest debriefs. Debriefs included review of physiologic goals for CPR and post-arrest care targets including hemodynamics. The primary outcome was survival to hospital discharge with favorable neurologic outcome (defined as Pediatric Cerebral Performance Category [PCPC] score of 1–3 or no change from baseline). The secondary outcome was survival to hospital discharge.

Data elements collected for the *ICU-RESUS* trial included subject demographics, arrest characteristics and post-cardiac arrest care data, and survival to hospital discharge and survival to hospital discharge with favorable neurologic outcome, per standard Utstein-style reporting [20]. Pediatric Risk of Mortality (PRISM) III score[21] was assessed 2–6 h prior to cardiac arrest. The vasoactive inotropic score (VIS) was calculated with the following equation: dopamine dose (μg/kg/min) + dobutamine dose (μg/kg/min) + 100 × epinephrine dose (μg/kg/min) + 10 × milrinone dose (μg/kg/min) + 10 000 × vasopressin dose (unit/kg/min) + 100 × norepinephrine dose (μg/kg/min) at 2 h prior to cardiac arrest, and 0–6 h and 6–24 h post-arrest [22, 23].

The *ICU-RESUS* parent study collected the highest and lowest invasive and non-invasive SBP and DBP were collected for each subject at 0–6 h and 6–24 h post-arrest[19], as was done in previous pediatric studies [12–14]. We focused primarily on early (0–6 h) hypotension for comparison to prior adult and pediatric studies [6–10, 12–14]. When available, intra-arrest blood pressure was measured via arterial catheters and analyzed in 30-s epochs for the first 10 min of CPR. Providers were trained to target previously reported intra-arrest blood pressure targets: intra-arrest DBP ≥ 25 mmHg for subjects < 1 year of age, and ≥ 30 mmHg for subjects ≥ 1 year [24, 25]. Post-arrest variables collected were highest and lowest temperature (°C), arterial PaO2 (mmHg), PCO2 (mmHg), pH, glucose, pulse oximetry (SpO2), and highest lactate in the 0–6 h and 6–24 h post-arrest intervals.

### Post-arrest blood pressure study

All patients from the *ICU-RESUS* trial were eligible for this study. We excluded patients without ROSC, who were treated with ECMO within 6 h of cardiac arrest, or who did not have blood pressure values available at 0–6 h post-arrest. The lowest SBP and DBP for each patient at 0–6 h and 6–24 h were percentile adjusted for age, sex, and height based on normative data of blood pressures in the healthy pediatric population [5]. All blood pressures were reviewed by trained staff to limit erroneous or artifactual values [19].

#### Identification of blood pressure thresholds associated with outcome

Using this cohort, we determined the threshold probability of survival to hospital discharge with favorable neurological outcome versus adjusted percentiles of lowest SBP and DBP at 0–6 h post-arrest. Probability splines were generated with logistic regression, controlling for illness category (medical cardiac, surgical cardiac, medical non-cardiac, surgical non-cardiac) and pre-arrest hypotension (defined as SBP < 5th percentile for age, sex, and height; mean arterial blood pressure < 5th percentile for age; or presence of vasopressor/inotropic support after volume expansion, excluding low-dose dopamine). A natural cubic spline with internal knots at the 10th, 50th, and 90th percentiles was used to accurately capture the relationship between post-arrest blood pressures and survival to hospital discharge with favorable neurologic outcome. We determined post-arrest threshold percentile cutoff points of SBP at 0–6 h and DBP at 0–6 h with the use AUROC curves (Additional file 1: Figure S1). Cutoffs were approximated to the nearest clinically relevant integer value. We generated and inspected natural cubic spline with internal knots at the 10th, 50th, and 90th percentiles to graphically depict the relationship between post-arrest blood pressures and survival to hospital discharge with favorable neurologic outcome. The spline curve provided further support for the choice of post-arrest threshold percentile cutoff points of SBP at 0–6 h and DBP at 0–6 h.

#### Association of blood pressure thresholds associated with outcome

The primary exposure was whether the lowest documented SBP or DBP at 0–6 and 6–24 h post-arrest met the prospectively identified SBP and DBP percentile thresholds. The primary outcome was survival to hospital discharge with favorable neurologic outcome and the secondary outcome was survival to hospital discharge. We hypothesized that patients with SBP and DBP above identified thresholds would have higher rates of survival to hospital discharge with favorable neurologic outcome.

For description and comparison of patient and event characteristics, subjects were dichotomized into groups based on prospectively identified SBP and DBP thresholds. Frequencies and percentages were used to summarize categorical variables, and medians and interquartile ranges were used to summarize continuous variables. Associations of covariates with post-arrest blood pressure above threshold were assessed with Fisher’s exact test for categorical variables and the Wilcoxon rank-sum test for ordinal variables. The relationship between vasoactive inotropic score at 6- and 24-h post-arrest and post-arrest SBP and DBP above threshold at 0–6 h post-arrest was assessed with the Wilcoxon rank-sum test. Vasoactive inotropic scores of zero were reported as ‘none’, 0–20, or > 20. Survival to hospital discharge and survival to hospital discharge with favorable neurologic outcome were summarized and assessed with Fisher’s exact test for categorical variables and the Wilcoxon rank-sum test for ordinal variables. These analyses were repeated at the 6–24 h post-arrest.

The association between SBP and DBP thresholds at 0–6 h post-arrest with (1) survival to hospital discharge with favorable neurologic outcome and (2) survival to hospital discharge were assessed using Poisson regression with robust error estimates, controlling for illness category and pre-arrest hypotension. These covariates were selected a priori by the writing group, prior to any data analysis [26], and were consistent among the ICU-RESUS planned secondary analyses [27, 28]. Analyses were repeated for lowest SBP and DBP categories at 6–24 h. To test for the impact of the original trial’s intervention, we added the variable of treatment category during the parent study (control group versus transition group versus *ICU-RESUS* bundle) and repeated all analyses.

#### Association of intra-arrest blood pressure and post-arrest blood pressure

Intra-arrest invasively measured blood pressure data were prospectively collected and analyzed as a component of the *ICU-RESUS* trial [18, 19, 25, 29]. We evaluated the association between intra-arrest mean DBP targets and post-arrest study SBP and DBP thresholds at 0–6 h post-arrest using Poisson regression with robust error estimates, controlling for illness category and pre-arrest hypotension. We evaluated the interaction between intra-arrest and post-arrest blood pressure thresholds on outcome.

Covariates for modeling were specified a priori. *P* values were reported based on a 2-sided alternative and considered statistically significant when less than 0.05. Analyses were performed using SAS software v9.4 (Cary, NC, USA).

## Results

**Fig. 1:**
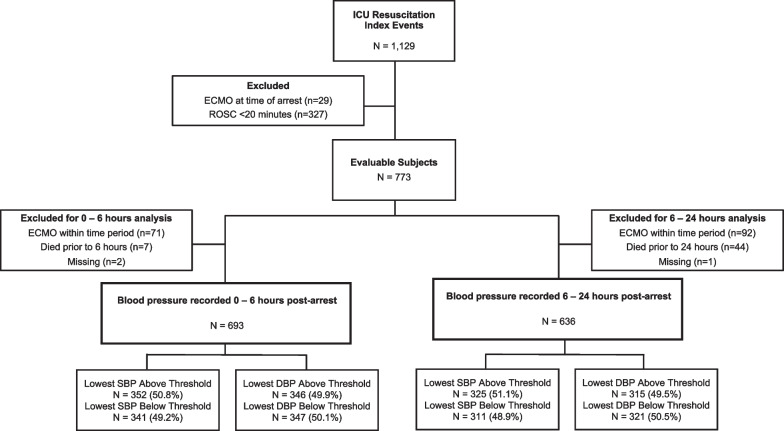
Consort Diagram

The cohort included 1,129 index events; 773 subjects were evaluable (Fig. 1). After excluding for initiation of ECMO and those who died during the time period, there were 693 analyzable subjects at 0–6 h post-arrest and 693 subjects at 6–24 h post-arrest. Of those that had BP data analyzed for 0–6 h post-arrest, 513 (74.0%) survived to hospital discharge, and 477 (68.8%) survived to hospital discharge with favorable neurologic outcome. Of the subjects with BP data for 6–24 h, 504 (79.3%) survived to hospital discharge, and 469 (73.7%) survived to hospital discharge with favorable neurologic outcome.

### Identification of systolic and diastolic blood pressure thresholds

**Fig. 2:**
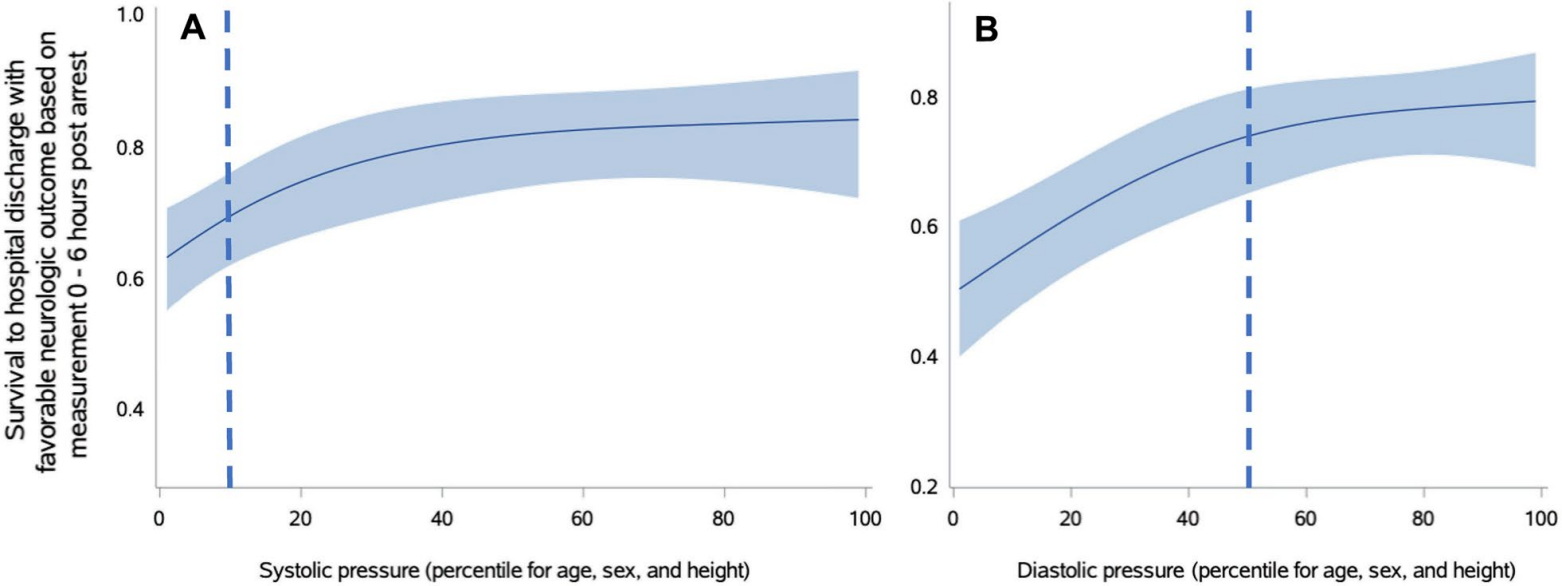
Splines of the association of age-, sex-, and height-adjusted systolic (Panel **A**) and diastolic (Panel **B**) blood pressure percentiles at 0–6 h post-arrest by survival to hospital discharge with favorable neurologic outcome controlling for pre-arrest hypotension and illness category. Light blue boundaries represent 95% confidence intervals. Dashed line represents identified blood pressure threshold

Optimal cutoffs on the AUROC curves were SBP at the 7th percentile adjusted for age, sex, and height and DBP at the 49th percentile adjusted for age, sex, and height. These were supported by splines. For clinical relevance, post-arrest lowest SBP above the 10th percentile at both 0–6 h was the threshold where the probability of survival to hospital discharge with favorable neurologic outcome plateaued. Lowest DBP above the 50th percentile at 0–6 h post-arrest was the threshold where the probability of survival to hospital discharge with favorable neurologic outcome plateaued (Fig. 2). These 0–6 h thresholds for lowest SBP of 10th percentile and lowest DBP of 50th percentile were used for subsequent analyses.

### Systolic blood pressure 0–6 h post-arrest

There were 352 subjects (50.8%) with the lowest SBP at 0–6 h post-arrest above the 10th percentile threshold (Table 1). These subjects received shorter durations of CPR; fewer doses of epinephrine during arrest; less frequently received sodium bicarbonate and calcium during arrest; and had lower VIS scores in the first 6 h post-arrest (Table 2). Fewer subjects with lowest SBP above threshold had hypotension as an immediate cause of arrest, but more had respiratory insufficiency.

**Table 1:** Demographics and pre-event characteristics by systolic and diastolic blood pressure thresholds at 0–6 h post-arrest

	Post-arrest systolic BP (0–6 h)^a^		*P* value	Post-arrest diastolic BP (0–6 h)^a^		*P* value
	> 10th percentile (*N* = 352)	< 10th percentile (*N* = 341)		> 50th percentile (*N* = 346)	< 50th percentile (*N* = 347)	
Demographics						
Age			< .001^c^			< .001^c^
≤ 1 month	27 (7.7%)	74 (21.7%)		63 (18.2%)	38 (11.0%)	
1 month–< 1 year	174 (49.4%)	133 (39.0%)		193 (55.8%)	114 (32.9%)	
1 year–< 8 years	108 (30.7%)	70 (20.5%)		67 (19.4%)	111 (32.0%)	
8 years–< 19 years	43 (12.2%)	64 (18.8%)		23 (6.6%)	84 (24.2%)	
Male	199 (56.5%)	175 (51.3%)	0.171^c^	191 (55.2%)	183 (52.7%)	0.542^c^
Race			0.017^c^			0.812^c^
White	156 (44.3%)	172 (50.4%)		158 (45.7%)	170 (49.0%)	
Black or African American	106 (30.1%)	68 (19.9%)		87 (25.1%)	87 (25.1%)	
Other	25 (7.1%)	22 (6.5%)		21 (6.1%)	26 (7.5%)	
Unknown or not reported	65 (18.5%)	79 (23.2%)		80 (23.1%)	64 (18.4%)	
Hispanic or Latino	51 (14.5%)	55 (16.1%)	0.396^c^	59 (17.1%)	47 (13.5%)	0.287^c^
Pre-existing medical conditions						
Respiratory insufficiency	319 (90.6%)	289 (84.8%)	0.020^c^	310 (89.6%)	298 (85.9%)	0.164^c^
Hypotension	154 (43.8%)	221 (64.8%)	< .001^c^	182 (52.6%)	193 (55.6%)	0.446^c^
Congestive heart failure	34 (9.7%)	28 (8.2%)	0.510^c^	31 (9.0%)	31 (8.9%)	1.000^c^
Pneumonia	58 (16.5%)	35 (10.3%)	0.019^c^	45 (13.0%)	48 (13.8%)	0.824^c^
Sepsis	44 (12.5%)	53 (15.5%)	0.274^c^	33 (9.5%)	64 (18.4%)	< .001^c^
Renal insufficiency	33 (9.4%)	46 (13.5%)	0.095^c^	21 (6.1%)	58 (16.7%)	< .001^c^
Congenital heart disease	175 (49.7%)	210 (61.6%)	0.002^c^	215 (62.1%)	170 (49.0%)	< .001^c^
Pre-event characteristics						
Illness category			< .001^c^			0.003^c^
Medical cardiac	76 (21.6%)	75 (22.0%)		89 (25.7%)	62 (17.9%)	
Medical non-cardiac	159 (45.2%)	111 (32.6%)		123 (35.5%)	147 (42.4%)	
Surgical cardiac	87 (24.7%)	129 (37.8%)		115 (33.2%)	101 (29.1%)	
Surgical non-cardiac or trauma	30 (8.5%)	26 (7.6%)		19 (5.5%)	37 (10.7%)	
Baseline Pediatric Cerebral Performance Category^b^			0.828^d^			< .001^d^
1—Normal	208 (59.1%)	206 (60.4%)		231 (66.8%)	183 (52.7%)	
2—Mild disability	59 (16.8%)	53 (15.5%)		55 (15.9%)	57 (16.4%)	
3—Moderate disability	46 (13.1%)	41 (12.0%)		38 (11.0%)	49 (14.1%)	
4—Severe disability	35 (9.9%)	38 (11.1%)		19 (5.5%)	54 (15.6%)	
5—Coma/vegetative state	4 (1.1%)	3 (0.9%)		3 (0.9%)	4 (1.2%)	

^a^Post-arrest systolic or diastolic thresholds defined as the minimum recorded systolic or diastolic blood pressure in the time period less than or equal to the 10th or 50th percentile for age, sex, and height, respectively

^b^Baseline pediatric cerebral performance category and functional status scale were evaluated prior to the event leading to hospitalization

^c^Fishers exact test

^d^Wilcoxon rank-sum test

**Table 2:** Event characteristics and outcomes by systolic and diastolic blood pressure thresholds at 0–6 h post-arrest

	Post-arrest systolic BP (0–6 h)^a^		*P* value	Post-arrest diastolic BP (0–6 h)^a^		*P* value
	> 10th percentile (*N* = 352)	< 10th percentile (*N* = 341)		> 50th percentile (*N* = 346)	< 50th percentile (*N* = 347)	
Interventions in place prior to event						
Vascular access	308 (87.5%)	320 (93.8%)	0.006^b^	301 (87.0%)	327 (94.2%)	0.001^b^
Arterial catheter	130 (36.9%)	174 (51.0%)	< .001^b^	152 (43.9%)	152 (43.8%)	1.000^b^
Central venous catheter	208 (59.1%)	232 (68.0%)	0.018^b^	220 (63.6%)	220 (63.4%)	1.000^b^
Vasoactive infusion	111 (31.5%)	167 (49.0%)	< .001^b^	138 (39.9%)	140 (40.3%)	0.938^b^
Invasive mechanical ventilation	224 (63.6%)	241 (70.7%)	0.052^b^	224 (64.7%)	241 (69.5%)	0.196^b^
Non-invasive ventilation	78 (22.2%)	63 (18.5%)	0.258^b^	75 (21.7%)	66 (19.0%)	0.397^b^
End-tidal CO_2_ monitoring	186 (52.8%)	226 (66.3%)	< .001^b^	193 (55.8%)	219 (63.1%)	0.053^b^
Immediate causes of event						
Arrhythmia	55 (15.6%)	47 (13.8%)	0.521^b^	49 (14.2%)	53 (15.3%)	0.748^b^
Cyanosis without respiratory decompensation	19 (5.4%)	9 (2.6%)	0.082^b^	18 (5.2%)	10 (2.9%)	0.128^b^
Hypotension	118 (33.5%)	196 (57.5%)	< .001^b^	139 (40.2%)	175 (50.4%)	0.008^b^
Respiratory decompensation	241 (68.5%)	192 (56.3%)	< .001^b^	225 (65.0%)	208 (59.9%)	0.182^b^
Vasoactive inotropic score (2 h prior to CPR)	0.0 [0.0, 0.8]	0.0 [0.0, 5.0]	< .001^c^	0.0 [0.0, 5.0]	0.0 [0.0, 3.0]	0.113^b^
Duration of CPR (minutes)^d^	3.0 [1.0, 6.0]	4.0 [2.0, 8.0]	0.005^c^	3.0 [1.0, 6.0]	4.0 [2.0, 8.0]	0.012^c^
Duration of CPR categories (minutes)^d^			0.129^b^			0.011^b^
< 6	247 (70.2%)	212 (62.2%)		244 (70.5%)	215 (62.0%)	
6–15	72 (20.5%)	83 (24.3%)		74 (21.4%)	81 (23.3%)	
16–35	26 (7.4%)	34 (10.0%)		24 (6.9%)	36 (10.4%)	
> 35	7 (2.0%)	12 (3.5%)		4 (1.2%)	15 (4.3%)	
CPR time^d^			0.904^b^			0.899^b^
Weekday	185 (52.6%)	184 (54.0%)		183 (52.9%)	186 (53.6%)	
Weeknight	57 (16.2%)	56 (16.4%)		55 (15.9%)	58 (16.7%)	
Weekend	110 (31.3%)	101 (29.6%)		108 (31.2%)	103 (29.7%)	
First documented rhythm			0.514^b^			0.219^b^
Pulseless electrical activity / asystole	132 (37.5%)	134 (39.3%)		124 (35.8%)	142 (40.9%)	
Ventricular fibrillation / tachycardia	17 (4.8%)	22 (6.5%)		17 (4.9%)	22 (6.3%)	
Bradycardia with poor perfusion	203 (57.7%)	185 (54.3%)		205 (59.2%)	183 (52.7%)	
Pharmacologic interventions during event						
Epinephrine	214 (60.8%)	267 (78.3%)	< .001^b^	226 (65.3%)	255 (73.5%)	0.021^b^
Minutes to first epinephrine bolus	1.0 [0.0, 2.0]	1.0 [0.0, 2.0]	0.817^c^	1.0 [0.0, 2.0]	1.0 [0.0, 2.0]	0.099^c^
Number of epinephrine boluses	2.0 [1.0, 3.0]	2.0 [1.0, 3.0]	0.713^c^	1.5 [1.0, 3.0]	2.0 [1.0, 3.0]	0.517^c^
Atropine	46 (13.1%)	33 (9.7%)	0.188^b^	46 (13.3%)	33 (9.5%)	0.122^b^
Calcium	60 (17.0%)	117 (34.3%)	< .001^b^	76 (22.0%)	101 (29.1%)	0.036^b^
Sodium bicarbonate	81 (23.0%)	135 (39.6%)	< .001^b^	95 (27.5%)	121 (34.9%)	0.040^b^
Vasopressin	5 (1.4%)	10 (2.9%)	0.199^b^	5 (1.4%)	10 (2.9%)	0.296^b^
Fluid bolus	56 (15.9%)	72 (21.1%)	0.079^b^	53 (15.3%)	75 (21.6%)	0.040^b^
Vasoactive-inotropic score						
Vasoactive inotropic score 6 h post-arrest			< .001^b^			0.006^b^
None	207 (58.8%)	133 (39.0%)		178 (51.4%)	162 (46.7%)	
1–20	126 (35.8%)	158 (46.3%)		146 (42.2%)	138 (39.8%)	
> 20	19 (5.4%)	50 (14.7%)		22 (6.4%)	47 (13.5%)	
Vasoactive inotropic score 24 h post-arrest			< .001^b^			0.188^b^
None	214 (60.8%)	153 (44.9%)		181 (52.3%)	186 (53.6%)	
1–20	123 (34.9%)	154 (45.2%)		146 (42.2%)	131 (37.8%)	
> 20	15 (4.3%)	34 (10.0%)		19 (5.5%)	30 (8.6%)	
Outcomes						
Survival to hospital discharge	289 (82.1%)	224 (65.7%)	< .001^b^	283 (81.8%)	230 (66.3%)	< .001^b^
Survival to hospital discharge with favorable neurologic outcome	269 (76.4%)	208 (61.0%)	< .001^b^	267 (77.2%)	210 (60.5%)	< .001^b^

^a^Post-arrest systolic or diastolic thresholds defined as the minimum recorded systolic or diastolic blood pressure in the time period less than or equal to the 10th or 50th percentile for age, sex, and height, respectively

^b^Fishers Exact test

^c^Wilcoxon rank-sum test

^d^CPR = Cardiopulmonary resuscitation

In univariate analysis, subjects with lowest SBP above threshold had higher rates of survival to hospital discharge with favorable neurologic outcome (76.4% vs 61.0%, *p* < 0.001) and survival to hospital discharge (82.1% vs. 65.7%, *p* < 0.001) (Table 2). In a multivariable model adjusting for illness category and pre-arrest hypotension, subjects with lowest SBP above the 10th percentile threshold were more likely to survive to hospital discharge with favorable neurologic outcome (RR 1.22 [95% CI 1.10, 1.35], *p* < 0.001) and survive to hospital discharge (RR 1.21 [95% CI 1.10, 1.33], *p* < 0.001) (Table 3). Controlling for *ICU-RESUS* treatment category in the initial interventional trial in addition to illness category and pre-arrest hypotension revealed similar results (Additional file 5: Table S4).

**Table 3:** Multivariable associations between systolic and diastolic pressure 0–6 h post arrest and survival

		Survival to hospital discharge with favorable neurologic outcome^a^		Survival to hospital discharge	
	Overall (*N* = 693)	Relative risk (95% CI)	*P* value	Relative risk (95% CI)	*P* value
Post-arrest systolic threshold (0–6 h)^c^			< .001		< .001
Above (> 10th percentile)	352 (50.8%)	1.22 (1.10, 1.35)		1.21 (1.10, 1.33)	
Below—inclusive (≤ 10th percentile)	341 (49.2%)	Reference		Reference	
Post-arrest diastolic threshold (0–6 h)^c^			< .001		< .001
Above (> 50th percentile)	346 (49.9%)	1.27 (1.15, 1.40)		1.23 (1.12, 1.34)	
Below—inclusive (≤ 50th percentile)	347 (50.1%)	Reference		Reference	
Controlling also for intra-arrest diastolic pressure (met target: yes, no)^b,c^(N = 227)					
Post-arrest systolic threshold (0–6 h)^c^			0.500		0.769
Above (> 10th percentile)	100 (44.1%)	1.06 (0.90, 1.25)		1.02 (0.88, 1.19)	
Below—inclusive (≤ 10th percentile)	127 (55.9%)	Reference		Reference	
Post-arrest diastolic threshold (0–6 h)^c^			0.586		0.636
Above (> 50th percentile)	121 (53.3%)	1.05 (0.89, 1.24)		1.04 (0.89, 1.20)	
Below—inclusive (≤ 50th percentile)	106 (46.7%)	Reference		Reference	
Interaction between intra- and post-arrest diastolic pressure (*N* = 227)			0.736		0.332
Above intra-arrest target and above post-arrest threshold	112 (16.2%)	1.21 (0.79, 1.87)		1.27 (0.81, 1.98)	
Above intra-arrest target and below post-arrest threshold	94 (13.6%)	1.19 (0.77, 1.84)		1.27 (0.80, 2.00)	
Below intra-arrest target and above post-arrest threshold	9 (1.3%)	1.33 (0.80, 2.19)		1.51 (0.92, 2.46)	
Below intra-arrest target and below post-arrest threshold	12 (1.7%)	Reference		Reference	

^a^Favorable neurologic outcome was defined as no more than moderate disability or no worsening from baseline Pediatric Cerebral Performance Category (PCPC). Baseline PCPC represents subject status prior to the event leading to hospitalization

^b^All models control for illness category and pre-arrest hypotension. Two, noted above, also control for whether the intra-arrest diastolic blood pressure target was met

^c^ Target intra-arrest diastolic pressure was ≥ 25 mmHg for subjects < 1 year old or ≥ 30 for subjects ≥ 1 year. Post-arrest systolic or diastolic thresholds

defined as the minimum recorded systolic or diastolic blood pressure in the time period ≤ the 10th or 50th percentile for age, sex, and height, respectively

### Diastolic blood pressure 0–6 h post-arrest

Half of the cohort (346 subjects, 49.9%) had lowest DBP above the 50th percentile threshold (Table 1). Subjects with lowest DBP above threshold more frequently had pre-existing congenital heart disease and medical-cardiac or surgical-cardiac illness categories (Table 1) and had lower vasoactive inotropic score in the first 6 h after arrest compared to those with lowest DBP below threshold (Table 2). Fewer subjects with lowest DBP above threshold had hypotension as the immediate cause of arrest (Table 2). Arrest duration was shorter in those with lowest DBP above threshold (3 vs. 4 min, *P* = 0.012) and these subjects less frequently received intra-arrest epinephrine, calcium, sodium bicarbonate, and fluid resuscitation.

In univariate analysis subjects with lowest DBP above threshold at 0–6 h post-arrest had higher rates of survival to hospital discharge with favorable neurologic outcome (77.2% vs. 60.5%, *p* < 0.001) and survival to hospital discharge (81.8% vs. 63.3%, *p* < 0.001; Table 2). In a multivariable model controlling for illness category and pre-arrest hypotension, lowest DBP above threshold was associated with higher rates of survival to hospital discharge with favorable neurologic outcome (aRR 1.27 [95% CI 1.15, 1.40], *p* < 0.001) and survival to hospital discharge (aRR 1.23 [95% CI 1.12, 1.34], *p* < 0.001; Table 3). Adding *ICU-RESUS* treatment category in the original study to the multivariate analysis yielded similar results (Additional file 6: Table S5).

### Systolic and diastolic blood pressure post-arrest 6–24 h

We compared groups with lowest SBP and lowest DBP above threshold to those below threshold at 6–24 h post-arrest and found similar patterns to the 0–6 h post-arrest (Additional file 2: Table S1). In contrast to findings at the 0–6-h post-arrest, subjects with lowest SBP and DBP above threshold at 6–24 h did not differ in intra-arrest administration of sodium bicarbonate compared to those with lowest SBP and DBP below threshold (Additional file 3: Table S2). Similar to 0–6 h post-arrest, lowest SBP and DBP above threshold at 6–24 h post-arrest were associated with higher rates of survival to hospital discharge with favorable neurologic outcome, and survival to hospital discharge (Additional file 3: Table S2), including after controlling for illness category and pre-arrest hypotension (Additional file 4: Table S3).

### Intra-arrest blood pressures and CPR quality measures and early post-arrest hemodynamics

Intra-arrest hemodynamics were available for analysis in 227 patients (Table 4). Compared to infants (≤ 1 year of age) with post-arrest SBP below threshold at 0–6 h, infants with post-arrest SBP above threshold had higher mean intra-arrest SBP. Similarly, infants with post-arrest DBP above threshold at 0–6 h had higher mean intra-arrest SBP and DBP, compared to infants below respective thresholds. A higher percentage of all patients (i.e., infants + older children) with post-arrest DBP above threshold at 0–6 h met intra-arrest age-based SBP targets than those with post-arrest DBP below threshold. An association between post-arrest SBP above threshold and intra-arrest SBP was not observed. There were no differences in measures of CPR quality including compression rate and chest compression fraction over the first 10 min of CPR between patients above and below post-arrest SBP or DBP thresholds. There was no interaction between intra-arrest and post-arrest DBP at 0–6 h on outcomes (Table 3). In this smaller cohort, when controlling for intra-arrest mean DBP threshold in addition to illness category and pre-arrest hypotension, post-arrest lowest DBP and SBP were not associated with survival to hospital discharge with favorable neurologic outcome or survival to hospital discharge (Table 3). Repeating this analysis on lowest SBP and DBP 6–24 h post-arrest, no significant associations were found (Additional file 4: Table S3).

**Table 4:** Intra-arrest hemodynamics and chest compression mechanics by systolic and diastolic blood pressure thresholds 0–6 h post

	Post-arrest systolic BP (0–6 h)^a^		*P* value	Post-arrest diastolic BP (0–6 h)^a^		*P* value
	> 10th percentile (*N* = 100)	< 10th percentile (*N* = 127)		> 50th percentile (*N* = 121)	< 50th percentile (*N* = 106)	
Intra-arrest event characteristics						
Average systolic blood pressure (mmHg)						
Age ≤ 1 year	82.0 [68.9, 103.3]	72.2 [58.4, 87.9]	0.006^c^	81.7 [64.8, 94.8]	67.2 [54.6, 82.3]	0.004^c^
Age > 1 year	101.6 [85.1, 124.6]	90.3 [69.3, 116.2]	0.205^c^	104.5 [95.1, 127.1]	90.3 [72.6, 115.4]	0.070^c^
Average systolic blood pressure ≥ 60 mmHg for age < 1 year or ≥ 80 mmHg for age ≥ 1 year	78 (78.0%)	87 (68.5%)	0.283^b^	96 (79.3%)	69 (65.1%)	0.009^b^
Average diastolic blood pressure (mmHg)						
Age ≤ 1 year	41.1 [32.3, 48.6]	36.3 [30.1, 47.0]	0.176^c^	41.5 [32.0, 49.9]	34.6 [29.5, 46.0]	0.026^c^
Age > 1 year	43.4 [37.5, 60.6]	44.5 [34.5, 54.7]	0.628^c^	53.8 [40.4, 63.7]	42.9 [34.5, 53.4]	0.052^c^
Average diastolic blood pressure ≥ 25 mmHg for age < 1 year or ≥ 30 mmHg for age ≥ 1 year	92 (92.0%)	114 (89.8%)	0.648^b^	112 (92.6%)	94 (88.7%)	0.363^b^
Average chest compression rate over the first 10 min	121.6 [112.7, 132.7]	124.6 [113.4, 132.8]	0.719^c^	121.97 [112.14, 132.00]	124.74 [116.30, 133.01]	0.256^c^
Average chest compression fraction ≥ 0.80	96 (96.0%)	117 (92.1%)	0.276^b^	116 (95.9%)	97 (91.5%)	0.268^b^
Average chest compression fraction over the first 10 min	1.0 [0.9, 1.0]	1.0 [0.9, 1.0]	0.078^c^	0.98 [0.95, 1.00]	0.98 [0.92, 1.00]	0.336^c^

^a^Post-arrest systolic or diastolic blood pressure thresholds defined as the minimum recorded systolic or diastolic blood pressure in the time period ≤ the 10th or 50th percentile for age, sex, and height, respectively

^b^Fishers Exact test

^c^Wilcoxon rank-sum test

## Discussion

In this study, we used a large prospectively collected dataset to define thresholds of lowest SBP and DBP at 0–6 h post-arrest that are associated with survival to hospital discharge with favorable neurologic outcome. After controlling for illness category and pre-arrest hypotension, absence of SBP below the 10th percentile and DBP below the 50th percentile during the first 6 h after ROSC were associated with higher rates of survival to hospital discharge with favorable neurologic outcome and survival to hospital discharge. This is the first study to prospectively identify post-arrest BP thresholds associated with outcomes and therefore establishes potential new targets for pediatric post-arrest care.

Hypotension is common after pediatric cardiac arrest and is associated with poor post-arrest outcomes [4, 11, 13, 14]. Prior work has focused on lower thresholds of hypotension, typically the 5th percentile for SBP for age, sex and height, which was selected a priori from normative blood pressure data in the general population [5]. In this study, we performed a robust analysis using a large prospectively collected cardiac arrest dataset to define lowest SBP and DBP thresholds during the first 6 h post-arrest based on probability of survival to hospital discharge with favorable neurologic outcome. These newly derived thresholds for lowest BP during the first 6 h post-arrest, the 10th percentile for SBP and 50th percentile for DBP, are higher than lowest BP thresholds in previous studies and the targets recommended in current guidelines [3]. Our findings suggest that we should target higher blood pressure than previously recommended to potentially impact outcomes. Furthermore, it is not clear if titrating to specific blood pressures impacts outcomes after pediatric or adult cardiac arrest. Although these findings are limited by available data which only had highest and lowest SBP and DBP, these newly derived thresholds could facilitate future evaluations for interventional studies.

Post-cardiac arrest blood pressure and the impact on outcome is complicated, as hypotension can be both a manifestation of the post-arrest myocardial dysfunction [30, 31] and systemic inflammation [32], which are highly inter-related and both modifiable. Myocardial dysfunction can be treated with vasoactive-inotropic infusions. Post-arrest systemic inflammation can be treated by steroids and/or induced hypothermia, and the associated vasodilatory shock can be treated with vasoconstrictor medications; importantly, we did not have time-synchronized post-arrest vasoactive and inotropic infusion rates available for further analyses of these relationships. Although the optimal medications and dosing for post-arrest myocardial dysfunction and post-arrest systemic inflammation are unknown, this study and others suggest that proactively maintaining adequate blood pressure is a reasonable therapeutic goal. Particularly, the importance of the association of early (0–6 h after arrest) hypotension and outcomes requires close clinical coordination to start monitoring and responding to blood pressure as quickly as possible after arrest to meet targets.

Several patient factors evaluated in this study were strongly associated with failure to exceed study BP thresholds. Not surprisingly, pre-arrest hypotension and vasoactive or inotropic support were associated with lowest SBP post-arrest below threshold. Pre-arrest renal insufficiency and congenital heart disease were associated with lowest SBP and lowest DBP post-arrest below threshold. While more granular analysis of these sub-populations was outside the scope of this study, these patients may require particular focus to understand their physiology and resuscitation, both intra- and post-arrest, to achieve optimal post-arrest outcomes. Proactive monitoring and targeted care for these high-risk groups may help decrease rates of hypotension and impact outcomes.

We found that subjects who had post-arrest DBP above threshold at 0–6 h were more likely to have met a priori intra-arrest SBP targets during the first 10 min of CPR. This association may be due to use of epinephrine or other vasoactive medications during resuscitation; though interestingly, this association did not persist for SBP thresholds and intra-arrest DBP or other CPR quality measures such as rate or compression fraction. Furthermore, the interactions between intra-arrest and post-arrest thresholds were not associated with outcomes, consistent with previous studies [33]. Previous studies show that intra-arrest DBP above target was associated with higher rates of survival to hospital discharge and with favorable neurologic outcome [24, 25]. While many would surmise that worse CPR quality, manifested as not achieving desired intra-arrest blood pressure targets, would be associated with worse post-arrest hypotension, we did not find this in our study. These findings highlight the complex and not well understood interactions between intra-arrest and post-arrest blood pressure. Notably, when we evaluated the association of post-arrest lowest SBP and DBP thresholds with outcomes and controlled for intra-arrest mean DBP, the association was no longer significant, suggesting that intra-arrest DBP confounds the relationship between the two. Further investigations of these interactions are needed.

This work has several limitations. The limitations of the primary study have been previously discussed [19]. Our analyses focused on the lowest DBP and SBP for each subject during each of the time intervals (0–6 h, and 6–24 h), consistent with most post-arrest investigations to date [12–14]. This may not reflect the total burden of hypotension during the time intervals [4]. Future work can focus on burden, as well as whether SBP or DBP thresholds are the main driver of outcomes. Mean arterial blood pressure (MAP) was not collected because normative data were not available at the time of the parent study design [18].

Additionally, post-arrest care was not standardized (though post-arrest goals were reviewed as part of the intervention), so sites monitored and treated post-arrest blood pressure at their discretion; we did not examine post-arrest myocardial dysfunction. As mentioned above, post-arrest time-synchronized VIS was not available for analysis. Variability of care among sites could impact both treatment of post-arrest hypotension and outcomes.

## Conclusions

We identified new thresholds of post-arrest blood pressure associated with survival to hospital discharge with favorable neurologic outcome and survival to hospital discharge: lowest SBP > 10th percentile for age and lowest DBP > 50th percentile for age. When controlling for illness category and pre-arrest hypotension, lowest DBP and SBP above these thresholds were associated with better outcomes. These findings continue to illustrate the importance of post-arrest blood pressure monitoring and management in efforts to improve outcomes from pediatric cardiac arrest.

### Supplementary Information

**Additional file 1.**.** Figure S1**: (A) Association of outcome of survival to hospital discharge with favorable neurologic outcome with systolic blood pressure 0-6 post-arrest. The optimal cut point is at the 7th percentile adjusted for age, sex, and height (sensitivity 66%, specificity 57%). Area under the curve is 0.64. (B) Association of outcome of survival to hospital discharge with favorable neurologic outcome with diastolic blood pressure 0-6 post-arrest. The optimal cut point is at the 49th percentile adjusted for age, sex, and height (sensitivity 60%, specificity 61%). Area under the curve is 0.64.

**Additional file 2. Supplemental Table 1.** Demographics and pre-event characteristics by systolic and diastolic blood pressure thresholds 6–24 h post-arrest.

**Additional file 3. Supplemental Table 2.** Event characteristics by diastolic and systolic blood pressure threshold 6–24 h post arrest.

**Additional file 4. Supplemental Table 3.** Multivariable associations between diastolic pressure 6–24 h post arrest and survival.

**Additional file 5. Supplemental Table 4.** Multivariable associations including treatment category between systolic and diastolic pressure 0–6 h post arrest and survival.

**Additional file 6. Supplemental Table 5.** Multivariable associations including treatment category between diastolic pressure 6–24 h post arrest and survival.

## Data Availability

The dataset from the ICU Resuscitation trial is available for public use via the National Institutes of Health (NIH) Biologic Specimen and Data Repository Information Coordinating Center (BioLINCC).

## Abbreviations

CPR: Cardiopulmonary resuscitation
BP: Blood pressure
DBP: Diastolic blood pressure
ICU: Intensive care unit
IHCA: In-hospital cardiac arrest
PCPC: Pediatric Cerebral Performance Category
PCAS: Post-cardiac arrest syndrome
SBP: Systolic blood pressure
VIS: Vasoactive-inotropic score
