# Supplementary material for: Identification of post-cardiac arrest blood pressure thresholds associated with outcomes in children: an ICU-Resuscitation study
Source: Crit Care. 2023 Oct 7;27:388. doi: 10.1186/s13054-023-04662-9 (PMC10559632; doi:10.1186/s13054-023-04662-9)
Supplement: Supplementary file 1 — Additional file 1.. Figure S1: (A) Association of outcome of survival to hospital discharge with favorable neurologic outcome with systolic blood pressure 0-6 post-arrest. The optimal cut point is at the 7th percentile adjusted for age, sex, and height (sensitivity 66%, specificity 57%). Area under the curve is 0.64. (B) Association of outcome of survival to hospital discharge with favorable neurologic outcome with diastolic blood pressure 0-6 post-arrest. The optimal cut point is at the 49th percentile adjusted for age, sex, and height (sensitivity 60%, specificity 61%). Area under the curve is 0.64. [file 13054_2023_4662_MOESM1_ESM.pdf]

Supplemental Figure 1

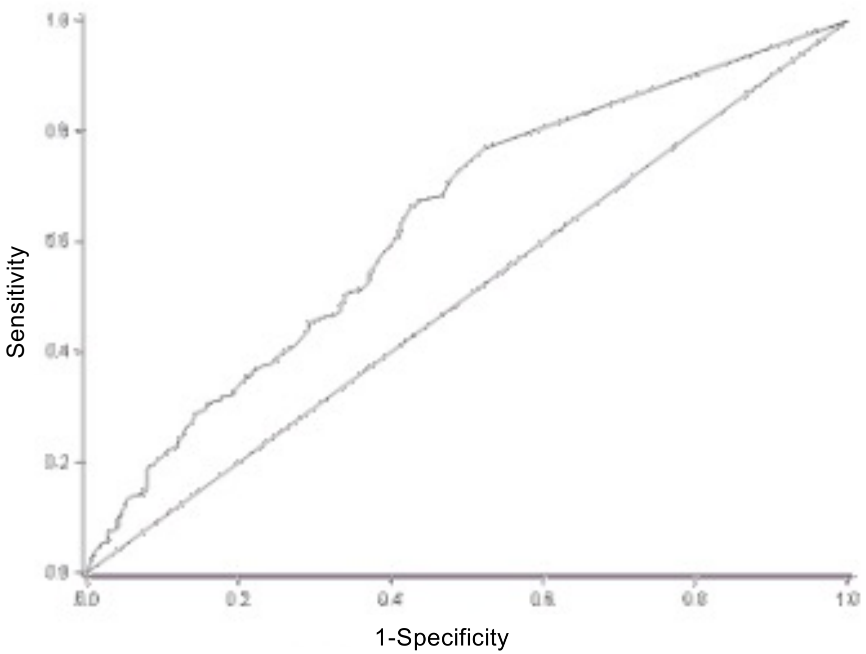

A. Association of outcome of survival to hospital discharge with favorable neurologic outcome with systolic blood pressure 0-6 post-arrest. **The optimal cut point is at the 7th percentile adjusted for age, sex and height (sensitivity 66%, specificity 57%).** Area under the curve is 0.64.

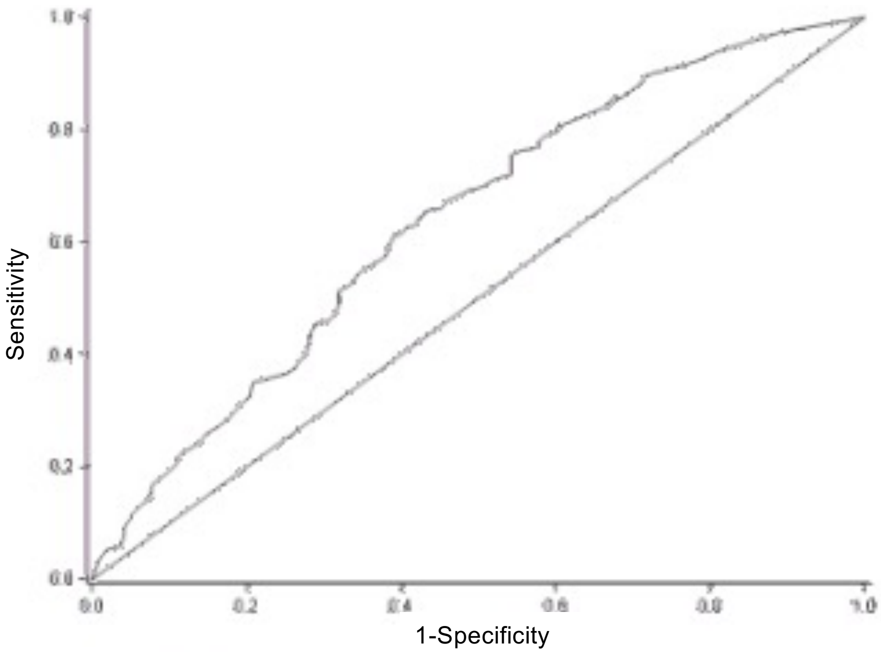

B. Association of outcome of survival to hospital discharge with favorable neurologic outcome with diastolic blood pressure 0-6 post-arrest. **The optimal cut point is at the 49th percentile adjusted for age, sex and height (sensitivity 60%, specificity 61%).** Area under the curve is 0.64.
