# Supplementary material for: Identification of post-cardiac arrest blood pressure thresholds associated with outcomes in children: an ICU-Resuscitation study
Source: Crit Care. 2023 Oct 7;27:388. doi: 10.1186/s13054-023-04662-9 (PMC10559632; doi:10.1186/s13054-023-04662-9)
Supplement: Supplementary file 2 — Additional file 2. Supplemental Table 1. Demographics and pre-event characteristics by systolic and diastolic blood pressure thresholds 6–24 h post-arrest. [file 13054_2023_4662_MOESM2_ESM.rtf]

Supplemental Table 1. Demographics and pre-event characteristics by systolic and diastolic blood pressure thresholds 6-24 hours post-arrest	
	Post-arrest systolic BP1
(6-24 hours)		Post-arrest diastolic BP1 
(6-24 hours)		
 	>10th percentile
(N = 325)	<10th percentile
(N = 311)	P-value	>50th percentile
(N = 315)	<50th percentile
(N = 321)	P-value	
Demographics							
  Age			<.0013			<.0013	
    ≤ 1 month	32 (9.8%)	60 (19.3%)		69 (21.9%)	23 (7.2%)		
    1 month - < 1 year	159 (48.9%)	132 (42.4%)		177 (56.2%)	114 (35.5%)		
    1 year - < 8 years	100 (30.8%)	62 (19.9%)		63 (20.0%)	99 (30.8%)		
    8 years - < 19 years	34 (10.5%)	57 (18.3%)		6 (1.9%)	85 (26.5%)		
  Male	176 (54.2%)	167 (53.7%)	0.9373	171 (54.3%)	172 (53.6%)	0.8743	
  Race			0.6923			0.9713	
    White	145 (44.6%)	149 (47.9%)		143 (45.4%)	151 (47.0%)		
    Black or African American	87 (26.8%)	76 (24.4%)		78 (24.8%)	85 (26.5%)		
    Other	24 (7.4%)	21 (6.8%)		21 (6.7%)	24 (7.5%)		
    Unknown or Not Reported	69 (21.2%)	65 (20.9%)		73 (23.2%)	61 (19.0%)		
  Hispanic or Latino	47 (14.5%)	53 (17.0%)	0.4423	55 (17.5%)	45 (14.0%)	0.2273	
Pre-existing medical conditions							
  Respiratory insufficiency	295 (90.8%)	265 (85.2%)	0.0373	277 (87.9%)	283 (88.2%)	1.0003	
  Hypotension	145 (44.6%)	185 (59.5%)	<.0013	163 (51.7%)	167 (52.0%)	1.0003	
  Congestive heart failure	29 (8.9%)	28 (9.0%)	1.0003	28 (8.9%)	29 (9.0%)	1.0003	
  Pneumonia	45 (13.8%)	40 (12.9%)	0.7283	36 (11.4%)	49 (15.3%)	0.1643	
  Sepsis	34 (10.5%)	46 (14.8%)	0.1203	28 (8.9%)	52 (16.2%)	0.0063	
  Renal insufficiency	34 (10.5%)	28 (9.0%)	0.5943	22 (7.0%)	40 (12.5%)	0.0233	
  Congenital heart disease	174 (53.5%)	188 (60.5%)	0.0923	211 (67.0%)	151 (47.0%)	<.0013	
Pre-event characteristics							
  Illness category			0.0073			<.0013	
    Medical cardiac	63 (19.4%)	78 (25.1%)		72 (22.9%)	69 (21.5%)		
    Medical non-cardiac	145 (44.6%)	99 (31.8%)		101 (32.1%)	143 (44.5%)		
    Surgical cardiac	91 (28.0%)	111 (35.7%)		126 (40.0%)	76 (23.7%)		
    Surgical non-cardiac or trauma	26 (8.0%)	23 (7.4%)		16 (5.1%)	33 (10.3%)		
  Baseline Pediatric Cerebral Performance Category2			0.9943			<.0013	
    1 - Normal	190 (58.5%)	183 (58.8%)		212 (67.3%)	161 (50.2%)		
    2 - Mild disability	57 (17.5%)	52 (16.7%)		53 (16.8%)	56 (17.4%)		
    3 - Moderate disability	39 (12.0%)	37 (11.9%)		28 (8.9%)	48 (15.0%)		
    4 - Severe disability	35 (10.8%)	35 (11.3%)		21 (6.7%)	49 (15.3%)		
    5 - Coma/vegetative state	4 (1.2%)	4 (1.3%)		1 (0.3%)	7 (2.2%)		
1 Post-arrest systolic or diastolic thresholds defined as the minimum recorded systolic or diastolic blood pressure in the time period less than or equal to the 10th or 50th percentile for age, sex, and height, respectively.
2 Baseline pediatric cerebral performance category were evaluated prior to the event leading to hospitalization.
3 Fishers exact test.
4 Wilcoxon rank-sum test.	
