# Supplementary material for: Identification of post-cardiac arrest blood pressure thresholds associated with outcomes in children: an ICU-Resuscitation study
Source: Crit Care. 2023 Oct 7;27:388. doi: 10.1186/s13054-023-04662-9 (PMC10559632; doi:10.1186/s13054-023-04662-9)
Supplement: Supplementary file 3 — Additional file 3. Supplemental Table 2. Event characteristics by diastolic and systolic blood pressure threshold 6–24 h post arrest. [file 13054_2023_4662_MOESM3_ESM.rtf]

Supplemental Table 2. Event characteristics by diastolic and systolic blood pressure threshold 6 - 24 hours post arrest	
	Post-arrest systolic BP
(6 - 24 hours)1		Post-arrest diastolic BP 
(6 - 24 hours)1		
 	>10th percentile 
(N = 325)	<10th  percentile
(N = 311)	P-value	>50th percentile
(N = 315)	<50th  percentile
(N = 321)	P-value	
Interventions in place prior to event							
  Vascular access	285 (87.7%)	292 (93.9%)	0.0092	278 (88.3%)	299 (93.1%)	0.0402	
  Arterial catheter	125 (38.5%)	148 (47.6%)	0.0202	154 (48.9%)	119 (37.1%)	0.0032	
  Central venous catheter	209 (64.3%)	194 (62.4%)	0.6222	207 (65.7%)	196 (61.1%)	0.2492	
  Vasoactive infusion	105 (32.3%)	137 (44.1%)	0.0032	139 (44.1%)	103 (32.1%)	0.0022	
  Invasive mechanical ventilation	202 (62.2%)	218 (70.1%)	0.0372	205 (65.1%)	215 (67.0%)	0.6172	
  Non-invasive ventilation	74 (22.8%)	60 (19.3%)	0.2872	69 (21.9%)	65 (20.2%)	0.6282	
  End-tidal CO2 monitoring	175 (53.8%)	192 (61.7%)	0.0452	179 (56.8%)	188 (58.6%)	0.6882	
Immediate Causes of Event							
  Arrhythmia	52 (16.0%)	44 (14.1%)	0.5802	43 (13.7%)	53 (16.5%)	0.3212	
  Cyanosis without respiratory decompensation	12 (3.7%)	13 (4.2%)	0.8392	13 (4.1%)	12 (3.7%)	0.8412	
  Hypotension	114 (35.1%)	162 (52.1%)	<.0012	135 (42.9%)	141 (43.9%)	0.8112	
  Respiratory decompensation	228 (70.2%)	180 (57.9%)	0.0012	202 (64.1%)	206 (64.2%)	1.0002	
Vasoactive inotropic score (2 hours prior to CPR)	0.0 [0.0, 2.0]	0.0 [0.0, 5.0]	0.0043	0.0 [0.0, 5.0]	0.0 [0.0, 0.0]	<.0013	
Duration of CPR (minutes)4	3.0 [1.0, 6.0]	4.0 [2.0, 7.0]	0.1063	3.0 [2.0, 6.0]	4.0 [1.0, 8.0]	0.1873	
Duration of CPR categories (minutes)4			0.5962			0.1852	
  <6	229 (70.5%)	204 (65.6%)		227 (72.1%)	206 (64.2%)		
  6-15	67 (20.6%)	72 (23.2%)		62 (19.7%)	77 (24.0%)		
  16-35	22 (6.8%)	27 (8.7%)		20 (6.3%)	29 (9.0%)		
  >35	7 (2.2%)	8 (2.6%)		6 (1.9%)	9 (2.8%)		
CPR time4			0.7782			0.2482	
  Weekday	178 (54.8%)	164 (52.7%)		174 (55.2%)	168 (52.3%)		
  Weeknight	51 (15.7%)	55 (17.7%)		57 (18.1%)	49 (15.3%)		
  Weekend	96 (29.5%)	92 (29.6%)		84 (26.7%)	104 (32.4%)		
First documented rhythm			0.5032			0.3132	
  Pulseless electrical activity / asystole	123 (37.8%)	117 (37.6%)		113 (35.9%)	127 (39.6%)		
  Ventricular fibrillation / tachycardia	14 (4.3%)	20 (6.4%)		14 (4.4%)	20 (6.2%)		
  Bradycardia with poor perfusion	188 (57.8%)	174 (55.9%)		188 (59.7%)	174 (54.2%)		
Pharmacologic interventions during event							
  Epinephrine	199 (61.2%)	229 (73.6%)	<.0012	207 (65.7%)	221 (68.8%)	0.4472	
  Minutes to first epinephrine bolus	1.0 [0.0, 2.0]	1.0 [0.0, 2.0]	0.5903	1.0 [0.0, 2.0]	1.0 [0.0, 2.0]	0.1783	
  Number of epinephrine boluses	1.0 [1.0, 2.0]	2.0 [1.0, 3.0]	0.1413	1.0 [1.0, 2.0]	2.0 [1.0, 3.0]	0.1833	
  Atropine	34 (10.5%)	39 (12.5%)	0.4562	34 (10.8%)	39 (12.1%)	0.6202	
  Calcium	62 (19.1%)	86 (27.7%)	0.0112	76 (24.1%)	72 (22.4%)	0.6402	
  Sodium bicarbonate	71 (21.8%)	110 (35.4%)	<.0012	84 (26.7%)	97 (30.2%)	0.3352	
  Vasopressin	4 (1.2%)	7 (2.3%)	0.3742	5 (1.6%)	6 (1.9%)	1.0002	
  Fluid bolus	52 (16.0%)	56 (18.0%)	0.5272	49 (15.6%)	59 (18.4%)	0.3982	
Vasoactive-inotropic score							
  Vasoactive inotropic score 6 hours post-arrest			<.0013			0.0123	
    None	179 (55.1%)	123 (39.5%)		143 (45.4%)	159 (49.5%)		
    1 - 20	130 (40.0%)	140 (45.0%)		149 (47.3%)	121 (37.7%)		
    > 20	16 (4.9%)	48 (15.4%)		23 (7.3%)	41 (12.8%)		
  Vasoactive inotropic score 24 hours post-arrest			<.0013			0.0053	
    None	196 (60.3%)	130 (41.8%)		151 (47.9%)	175 (54.5%)		
    1 - 20	117 (36.0%)	144 (46.3%)		147 (46.7%)	114 (35.5%)		
    > 20	12 (3.7%)	37 (11.9%)		17 (5.4%)	32 (10.0%)		
Outcomes							
  Survival to hospital discharge	274 (84.3%)	230 (74.0%)	0.0022	266 (84.4%)	238 (74.1%)	0.0022	
  Survival to hospital discharge with favorable neurologic outcome	258 (79.4%)	211 (67.8%)	0.0012	255 (81.0%)	214 (66.7%)	<.0012	
1 Post-arrest systolic or diastolic thresholds defined as the minimum recorded systolic or diastolic blood pressure in the time period less than or equal to the 10th or 50th percentile for age, sex, and height, respectively.
2 Fishers Exact Test
3 Wilcoxon rank-sum test.
4 CPR = Cardiopulmonary resuscitation.	
