# Supplementary material for: Identification of post-cardiac arrest blood pressure thresholds associated with outcomes in children: an ICU-Resuscitation study
Source: Crit Care. 2023 Oct 7;27:388. doi: 10.1186/s13054-023-04662-9 (PMC10559632; doi:10.1186/s13054-023-04662-9)
Supplement: Supplementary file 4 — Additional file 4. Supplemental Table 3. Multivariable associations between diastolic pressure 6–24 h post arrest and survival. [file 13054_2023_4662_MOESM4_ESM.rtf]

Supplemental Table 3. Multivariable associations between diastolic pressure 6 - 24 hours post arrest and survival	
	Survival to hospital discharge with favorable neurologic outcome1	Survival to hospital discharge	
 	Overall (N = 636)	Relative risk (95% CI)	P-value	Relative risk (95% CI)	P-value	
Post-arrest systolic threshold (6 - 24 hours)3			0.006		0.012	
  Above (>10th percentile)	325 (51.1%)	1.14 (1.04, 1.25)		1.11 (1.02, 1.20)		
  Below (<10th percentile)	311 (48.9%)	Reference		Reference		
Post-arrest diastolic threshold (6 - 24 hours)3			<.001		0.001	
  Above (>50th percentile)	315 (49.5%)	1.21 (1.10, 1.34)		1.14 (1.05, 1.24)		
  Below (<50th percentile)	321 (50.5%)	Reference		Reference		
Controlling also for intra-arrest diastolic pressure (met target: yes, no)2,3(N = 206)						
  Post-arrest systolic threshold (6 - 24 hours)3			0.387		0.458	
    Above (>10th percentile)	102 (49.5%)	1.07 (0.92, 1.24)		1.05 (0.92, 1.21)		
    Below (<10th percentile)	104 (50.5%)	Reference		Reference		
  Post-arrest diastolic threshold (6 - 24 hours)3			0.323		0.257	
    Above (>50th percentile)	122 (59.2%)	1.09 (0.92, 1.28)		1.09 (0.94, 1.26)		
    Below (<50th percentile)	84 (40.8%)	Reference		Reference		
  Interaction between intra- and post-arrest diastolic pressure (N = 206).			0.244		<.001	
    Above intra-arrest target and above post-arrest threshold	112 (17.6%)	1.38 (0.79, 2.39)		1.44 (0.81, 2.54)		
    Above intra-arrest target and below post-arrest threshold	75 (11.8%)	1.32 (0.75, 2.32)		1.39 (0.78, 2.47)		
    Below intra-arrest target and above post-arrest threshold	10 (1.6%)	1.59 (0.90, 2.81)		1.80 (1.02, 3.16)		
    Below intra-arrest target and below post-arrest threshold	9 (1.4%)	Reference		Reference		
1 Favorable neurologic outcome was defined as no more than moderate disability or no worsening from baseline Pediatric Cerebral Performance Category (PCPC). Baseline PCPC represents subject status prior to the event leading to hospitalization.
2 All models control for illness category and pre-arrest hypotension. Two, as noted above, also control for whether the intra-arrest diastolic blood pressure target was met.
3 Target intra-arrest diastolic pressure was ≥ 25 mmHg for subjects < 1 year old or ≥ 30 for subjects ≥ 1 year.  Post-arrest systolic or diastolic thresholds 
defined as the minimum recorded systolic or diastolic blood pressure in the time period ≤ the 10th or 50th percentile for age, sex, and height, respectively.
	
